# Orthotopic liver transplantation for Management of a Giant Liver Hemangioma: a case report and review of literature

**DOI:** 10.1186/s12893-020-00801-z

**Published:** 2020-06-29

**Authors:** Hesameddin Eghlimi, Peyman Arasteh, Nazanin Azade

**Affiliations:** Shiraz Transplant Research Center, Shiraz University of Medical Sciences, Shiraz, Iran

**Keywords:** Hepatic Hemangioma, Liver transplantation, Benign liver neoplasms, Cavernous Hemangioma

## Abstract

**Background:**

Hepatic hemangioma (HH) is the most common benign tumor of the liver. In special conditions such as rapidly growing tumors, persistent pain, hemorrhage and when pressure effect on adjacent organs exist treatment is indicated. Surgical management is the most common treatment for HH.

**Case presentation:**

A 38-year-old male patient was diagnosed with HH for 7 years. The initial presentation of the mass was progressive abdominal distention causing early satiety, gastro-esophageal reflux disease, vomiting, dysphagia and weight loss. Later, the patient developed bilateral lower extremity edema. Imaging with computed tomography (CT scan) showed a large mass measuring 32.4*26*3.1 cm which was considered unresectable. The patient underwent a deceased donor liver transplantation. The excised mass was 9 kg. After nine days of hospitalization the patient was discharged in good condition. Three months later, the patient was admitted due to fever and cytomegalovirus infection for which he received intravenous ganciclovir and was discharged. In the latest follow-up the patient had no liver or kidney dysfunction eight months after the transplantation.

**Conclusion:**

With appropriate patient selection, liver transplantation can be considered as a treatment option for patients with huge HHs which are life-threatening and surgically unresectable.

## Background

Hepatic Hemangioma (HH) is the most common benign tumor of the liver. The tumor has a vascular nature and is usually solitary and small in size. The majority of HHs originate from the right hepatic lobe. Classically HHs are not clinically symptomatic and are incidental findings in imaging studies [1]. No definite genetic background has been suggested for the occurrence of HH; however few cases of familial hepatic hemangiomas have been described in literature [2]. A well-established gender disparity with female to male ration of 5:1 is reported for the tumor [3]. Estrogen therapy and pregnancy are the major causes promoting tumor growth in HHs, highlighting the role of female sex hormones in the pathogenesis of the tumor [4]. Adulthood is the usual period of presentation, with the average age of diagnosis varying from 30 to 50 years old [5]. HH tumors are mostly asymptomatic; however larger tumors present with abdominal discomfort and rarely cause jaundice, high cardiac output heart failure, hemorrhage and consumptive coagulopathy, a syndrome known as Kasabach-Merritt syndrome (KMS). Treatment of HH is only indicated in special cases [1, 6]. Herein, we present a case of a 9 kg giant HH that underwent liver transplantation and evaluate existing literature.

## Case presentation

A 38-year-old patient was under conservative follow-up for a huge HH for 7 years. At the time of presentation, the patient had noticed gradual abdominal distention and epigastric discomfort for which he sought medical consultation. He was previously healthy and had no significant past medical or family history of any significant disease. Imaging with ultrasonography and abdominal 4-phase CT scan were in favor of a hemangioma probably originating from the fifth and eighth right liver lobe, initially measuring 12*10*1.5 cm.

**Fig. 1:**
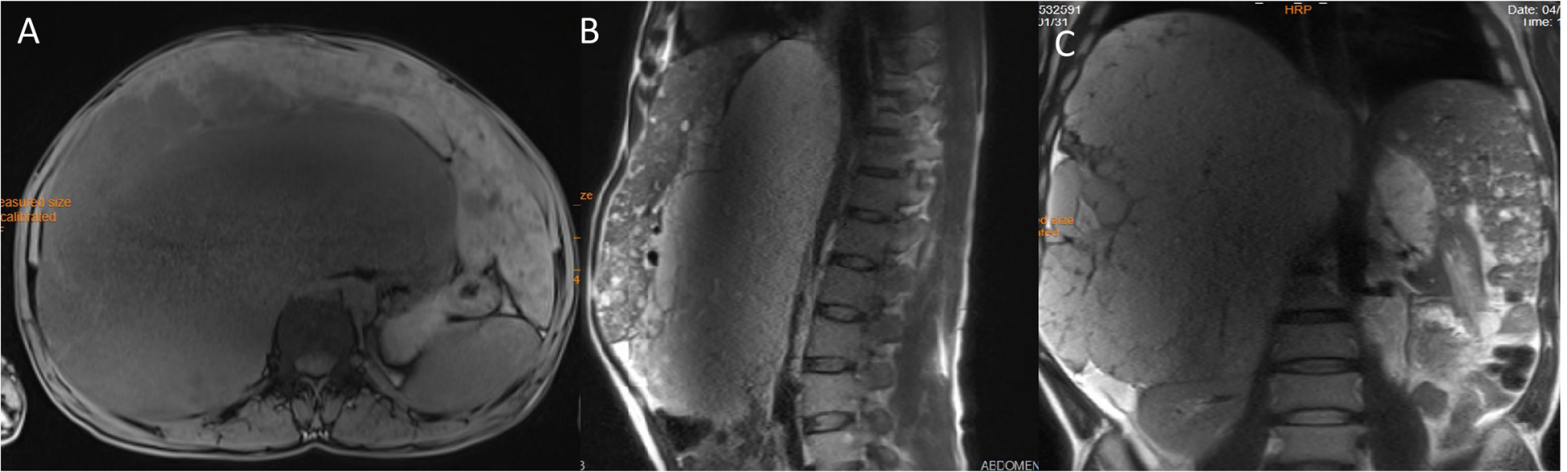
T1-weighted abdominal MRI showing a huge heterogeneous mass originating from right liver lobe in axial, sagittal and coronal views (**a**, **b** and **c**, respectively) with nodular discontinuous enhancement after gadolinium injection (**b**, **c**)

At the time of diagnosis, his management plan included a biannual follow-up of the mass via imaging studies including abdominal ultrasonography and magnetic resonance imaging (MRI). During the next 7 years the mass was growing in size and the patient complained of exacerbation of abdominal discomfort, early satiety, vomiting and bilateral lower extremity edema. Due to mass effect of the HH, he developed disturbing gastro-esophageal reflux disease causing significant weight loss and dysphagia to solid foods, for which an upper gastrointestinal tract endoscopy was performed. The study revealed severe esophagitis and stricture of the lower portion of the esophagus with hiatal hernia. The last abdominal MRI showed a heterogeneous solid mass measuring 32.4*26*3.1 cm in size originating from the right liver lobe. The mass had displaced the right kidney downwards and the right diaphragm upwards, however it had not caused any pressure effect on the intra or extra hepatic bile ducts. The IVC was also compressed, causing venous stasis in the lower extremities (Fig. 1).

Ultrasonography of the portal system showed pressure effect of the mass causing deviation of the portal vein and hepatic artery to the left sub-diaphragmatic aspect of the abdomen with mild portal hypertension and small amount of ascitic fluid. However, he had no esophageal varices reported in esophagoscopy. Despite the huge mass size, the patient did not develop KMS and had a relatively normal liver function test until the transplantation, which was as followed: AST = 45 IU/L (normal range: 5–42 IU/L), ALT = 34 IU/L (normal range: 5–37 IU/L), ALP = 256 IU/L (normal range: 50–275 IU/L) with total and direct bilirubin of 0.9 mg/DL (normal range 0–1 mg/DL) and 0.3 mg/DL (normal range: 0–0.35 mg/DL); respectively. He also had a platelet count of 256.000 (normal range: 141.000–356.000) and prothrombin time of 15.6 (normal range: 11–12.5) seconds. His echocardiography was also normal with an ejection fraction of 55%.

Later, due to patient’s extreme discomfort and risk of rupture and hemorrhage, a multi-disciplinary team decided to put the patient on the liver transplant waiting list. After 12 months, the patient underwent whole organ liver transplantation using a cadaver graft. During laparotomy, numerous collateral abdominal veins and approximately 400 cc of ascitic fluid were observed.

**Fig. 2:**
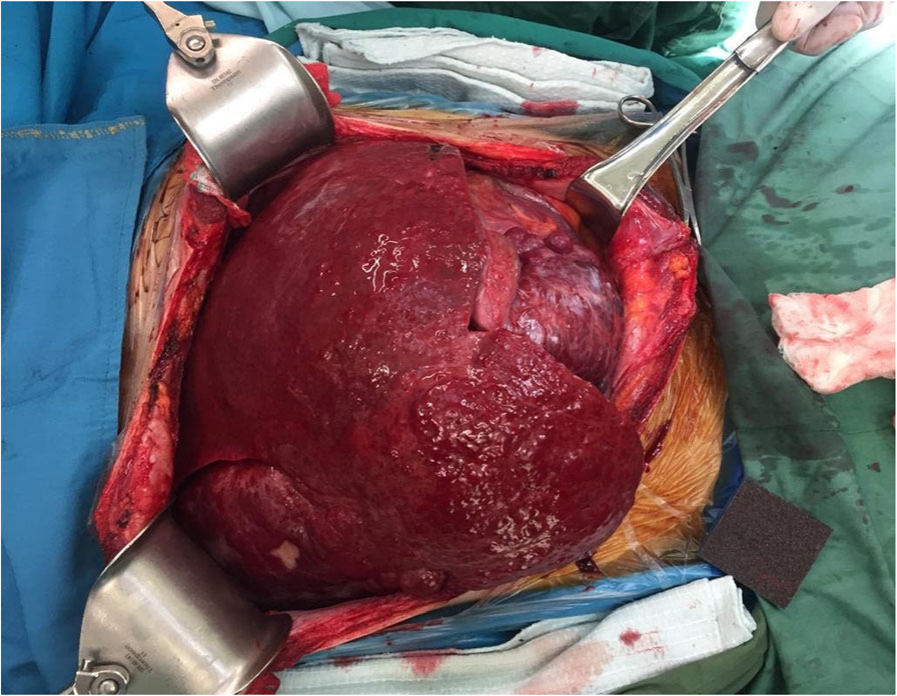
Gross pathology of the native liver showing a huge inhomogeneous well-circumcised sub-capsular mass weighing 9 kgs which occupied the whole abdomen

The native liver was dissected using a traditional hepatectomy technique and the estimated bleeding during hepatectomy was 1000 cc. Duct to duct anastomosis was done with 1000 cc bleeding after reperfusion. Fluid transfusion during surgery included 2500 cc of crystalloid fluid and 20 mg of albumin before and after declamping. He also received a transfusion of 4 units of packed red blood cells during the operation. The surgery lasted for 330 min and the patient was transferred to the intensive care unit with no acute complication. The explanted liver weighed 9 kg (Fig. 2).

**Fig. 3:**
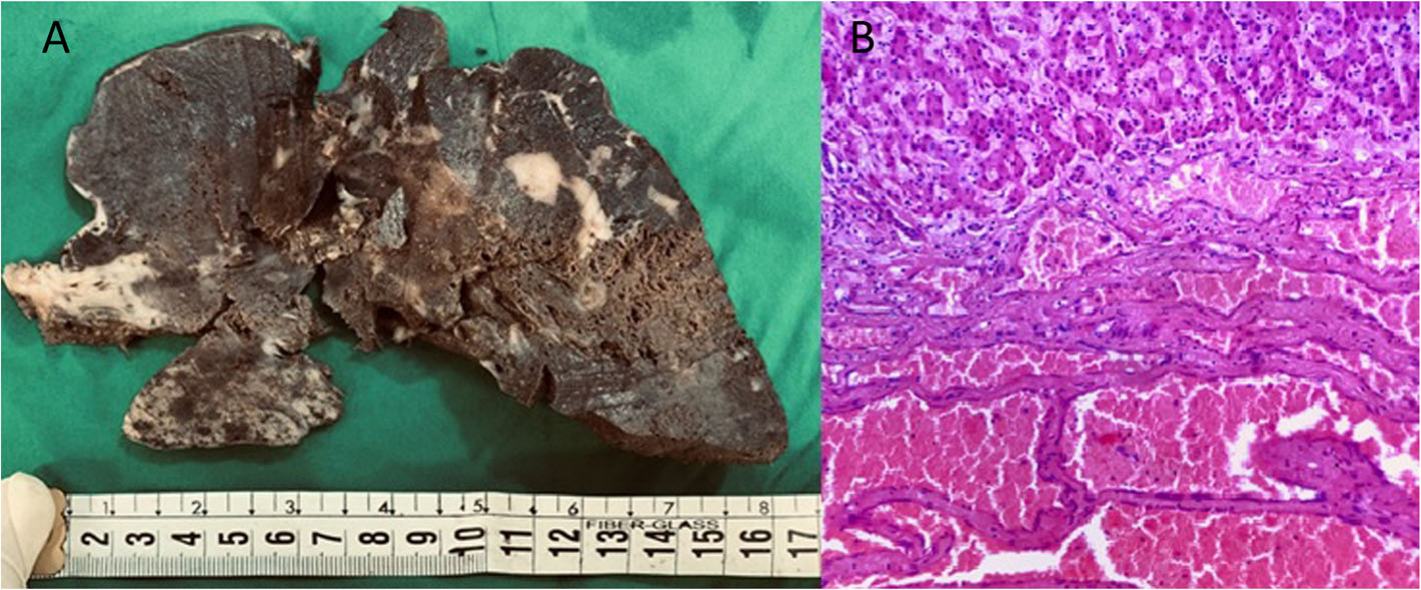
Cut section of liver with multiple diffuse ill-defined spongy brown masses (**a**) and numerous dilated blood vessels adjacent to hepatocytes (H&E × 200) (**b**)

During pathologic examination, serial sections showed multiple infiltrative masses with spongy microcystic surface occupying the whole liver (Fig. 3a).

Microscopic examination revealed dilated vascular spaces, located between hepatocytes, lined by endothelial cells and containing red blood vessels. There was no significant atypia (Fig. 3b).

Patient was discharged after 9 days with an immunosuppressive regimen of Tacrolimus (Prograf® 2 mg Q12H) and Mycophenolate (Myfortic® 360 mg Q12H). Three months post-surgery, the patient was admitted with fever and had a positive PCR for cytomegalovirus. During admission the patient was given intravenous gancyclovir (Valcyte® 350 mg Q24H, 10 days) and was discharged after a 7 day admission period with good conditions.

## Discussion and conclusion

Hepatic hemangioma (HH) is a common benign liver neoplasm. Due to different prognosis and complications of liver masses, HH should be differentiated from other benign or malignant lesions using various imaging modalities and other diagnostic methods [7]. Most HHs remain asymptomatic during a persons’ lifetime and usually do not have the potential for malignant transformation. Considering these, most HHs do not require medical intervention and annual or biannual imaging follow-up is sufficient in the majority of cases. Treatment is only indicated for rapidly growing tumors, persistent pain, hemorrhage and when pressure effect on adjacent organs and vessels exists, which may results in symptoms such as Budd-Chiari syndrome, jaundice and lower extremity edema. KMS is also an indication to seek treatment, the syndrome is characterized by thrombocytopenia, coagulopathy and microangiopathic hemolytic anemia [1]. Spontaneous or trauma induced bleeding from the tumor, is a rare but potentially fatal complication of HH which needs emergent laparotomy [6].

Up to this date, no medication has been proposed as a definite choice for medical treatment of HH. Some previous studies have reported promising results with medical management of HHs using bevacizumab, sorafenib, interferon and combination of sirulimus with high dose propranolol, however more studies are needed to support these findings [8–11].

In rare cases, patients with huge HH undergo liver transplantation. Indications for liver transplantation include huge masses compromising liver function, KMS and inoperable life threatening huge masses [12]. Up to this date and to the best of the authors’ knowledge, 20 liver transplantation for huge HH have been reported in 15 studies, using both living and deceased donor liver transplantation [13–27]. The youngest patient was a 4 week old infant [15] and the oldest to have liver transplantation was 51 years old [27]. Most transplants have been done due to KMS (*n* = 9) [13–16, 20–22, 24, 25]. Other causes included diffuse mass or rapid growth with imminent rupture (*n* = 4) [19, 21, 23, 26], respiratory distress (*n* = 4) [14, 21, 25, 26], rupture (*n* = 2) [16, 24], pain/discomfort (*n* = 3) [16, 17] and bleeding (*n* = 1) [17]. A summary of existing literature is presented in Table 1.

**Table 1:** Existing literature on liver transplantation in hepatic hemangioma

Report no.	Author (year)	Age (yrs)/sex	Graft Type	Follow-up	Cause of Tx	Complications	Condition/cause of death
1.	Klompmaker et al. (1989) [13]	28/Male	Whole	3 years	KMS	Uneventful	Alive
2.	Mora, et al. (1995) [14]	42/Female	Whole	16 days postop	KMS, respiratory distress	NA	Alive
3.	Tepetes et al. (1995) [15]	4wks/Male	Whole	8 days	KMS	Graft mal-function, intraventricular hemorrhage	Died, graft mal-function
4.	Brouwers et al. (1997) [16]	NM	Whole	1. 1 month 2. 1 year 3. 4 years 4. 9 years	Pain (*n* = 2), rupture (*n* = 1), KMS (*n* = 1)	1. Rejection, bile leakage & pleural effusion; 2. cytomegalovirus pneumonia, duodenal ulcer, steroid diabetes, peripheral nerve palsy & *Strongyloides stercoralis* infection; 3. uneventful	1. died Others alive
5.	Chui et al. (1996) [17]	1. 33/Female 2. 43/Female	Whole	1. 18 months 2. 14 months	1. Bleeding 2. Abdominal discomfort	1. Massive hemorrhage during surgery, ischemic graft with malfunction, acute renal failure, second transplantation was done; 2. Uneventful	Both alive
6.	Longeville et al. (1997) [18]	47/Male	Whole	12 months	KMS	Post-transplantation internal hemorrhage	Alive
7.	Russo et al. (1997) [19]	43/Female	Whole	14 days postop	Huge mass	NA	Alive
8.	Kumashiro, et al. (2002) [20]	48/Female	Posterior lobe	15 days postop	KMS, acute liver failure	Massive hemorrhage during operation due to KMS, uneventful post-operation course	Alive
9.	Ferraz et al. (2004) [21]	28/Female	Whole	30 months	KMS, respiratory distress, huge mass size	One episode of acute rejection treated with corticosteroid pulse	Alive
10.	Meguro et al. (2008) [22]	45/Female	Left Lobe	10 months	KMS	Massive hemorrhage during operation, acute rejection and small for size graft syndrome, sepsis	Alive
11.	Zhong et al. (2014) [23]	27/Female	Right lobe	50 months	Huge mass	Two episodes of acute rejection	Alive
12.	Vagefi et al. (2011) [24]	39/Female	Whole	NM	Rupture, KMS	Uneventful	Alive
13.	Yildiz et al. (2014) [25]	44/Female	Whole	1 month	KMS, respiratory distress	Uneventful	Alive
14.	Lange et al. (2015) [26]	46/Female	Whole	7 wks	Huge mass causing portal vein thrombosis, ascites, DVT & PTE	Uneventful	Alive
15.	Lee et al. (2017) [27]	51/Female	Modified Right Lobe	16 months	Rapid Growth	Uneventful	Alive

*KMS* Kasabach-Merritt Syndrome; *POSTOP* Postoperative; *TAE* Transcatheter angiographic embolization; *NM* Not mentioned; *NA* Not accessible manuscript; *DVT* Deep vein thrombosis; *PTE* Pulmonary thromboembolism; *TX* transplantation

Currently the most common treatment approach, especially with huge HHs is surgical intervention. Treatment options for huge cavernous HHs include surgical resection, transcatheter angiographic embolization (TAE), radiofrequency ablation, radiotherapy and in some cases orthotopic liver transplantation, as discussed earlier [1]. Minimally invasive techniques have been more frequently applied in recent years. The TAE method is done via catheterization of femoral artery and getting access to the hepatic artery to discover the tumor’s feeding arteries. The feeding arteries are then embolized by using an embolic agent. Arterial embolization is usually left for tumors with a definite arterial supply and is usually indicated prior to surgical resection of inoperable lesions to reduce the tumor’s size, facilitating the surgery [28]. Successful treatment of HH with TAE without surgery has also been reported [29]. Radiofrequency ablation uses high frequency current passing through an electrode which creates a small area of heat targeting the lesion. This method is either applied percutaneously or via laparoscopy and laparotomy. For most cases, significant symptom relief is achieved by RF-ablation. Due to difficult application of radiofrequency ablation technique for lesions larger than 10 cm, patients with larger HH tumors do not efficiently benefit from this method [30, 31]. Radiotherapy is a less frequently applied method for management of HH [32].

Surgical treatment of HH is considered for patients with severe symptoms affecting lifestyle, those suspicious of malignancy and huge tumors as they have an increased risk of rupture and bleeding [6]. Yet the optimal surgical approach still remains to be controversial. Surgeons may consider either segmental resection or enucleation of the tumor based on the location of the lesion. One meta-analysis conducted in 2016 reported that tumor tissue removal by both techniques can be safe and efficient; however due to decreased amount of intraoperative bleeding and a better preservation of normal hepatic tissue the enucleation method is the preferred surgical procedure [33].

## Conclusion

With appropriate patient selection, liver transplantation can be considered as a treatment option for patients with huge hemangiomas of the liver when other treatment options have failed or are not indicated.

## Data Availability

Not applicable.

## Abbreviations

CT scan: Computed tomography scan
HH: Hepatic hemangioma
KMS: Kasabach-meritt syndrome
MRI: Magnetic resonance imaging
TAE: Transcatheter angiographic embolization
